# ƩS COVID-19 is a rapid high throughput and sensitive one-step quadruplex real-time RT-PCR assay

**DOI:** 10.1038/s41598-024-71705-8

**Published:** 2024-09-04

**Authors:** Ekasit Kowitdamrong, Sasiprapa Anoma, Thitiya Loykaew, Pokrath Hansasuta, Parvapan Bhattarakosol

**Affiliations:** 1 Department of Microbiology, Faculty of Medicine, Chulalongkorn University, Bangkok, 10330 Thailand; 2 Center of Excellence in Applied Medical Virology, Chulalongkorn University, Bangkok, 10330 Thailand; 3 Department of Microbiology, King Chulalongkorn Memorial Hospital, Thai Red Cross, Bangkok, 10330 Thailand

**Keywords:** SARS-CoV-2, COVID-19, Real time RT-PCR, Rapid, High throughput, SARS-CoV-2, Infectious-disease diagnostics

## Abstract

Real-time reverse transcription polymerase chain reaction (RT-PCR), a standard method recommended for the diagnosis of coronavirus disease 2019 (COVID-19) requires 2–4 h to get the result. Although antigen test kit (ATK) is used for COVID-19 screening within 15–30 min, the drawback is its limited sensitivity. Hence, a rapid one-step quadruplex real-time RT-PCR assay: termed ƩS COVID-19 targeting ORF1ab, ORF3a, and N genes of SARS-CoV-2; and Avocado sunblotch viroid (ASBVd) as an internal control was developed. Based on strategies including designing high melting temperature primers with short amplicons, applying a fast ramp rate, minimizing hold time, and reducing the range between denaturation and annealing/extension temperatures; the assay could be accomplished within 25 min. The limit of detection of ORF1ab, ORF3a, and N genes were 1.835, 1.310, and 1 copy/reaction, respectively. Validation was performed in 205 combined nasopharyngeal and oropharyngeal swabs. The sensitivity, specificity, positive predictive value, and negative predictive value were 92.8%, 100%, 100%, and 97.1%, respectively with 96.7% accuracy. Cohen’s Kappa was 0.93. The newly developed rapid real-time RT-PCR assay was highly sensitive, specific, and fast, making it suitable for use as an alternative method to support laboratory diagnosis of COVID-19 in outpatient and emergency departments.

## Introduction

Coronavirus disease 2019 (COVID-19) is caused by a novel betacoronavirus, severe acute respiratory syndrome coronavirus 2 (SARS-CoV-2), first reported in Wuhan, Hubei, China in late 2019. COVID-19 rapidly spread and resulted in a pandemic with 772,166,517 confirmed cases and 6,981,263 cumulative deaths globally^1^. Even though many countries have dispensed an emergency use authorization (EUA) version of COVID-19 vaccines to their people, this measure could not terminate its threat to humans. The virus evolves continuously and evades host immunity with many variants arising such as alpha (B.1.1.7), beta (B.1.351), gamma (P.1), delta (B.1.617.2); and omicron (B.1.1.529) and its subvariants at the present^2,3^.

SARS-CoV-2 is classified in the Family *Coronaviridae*, Genus *Betacoronavirus*. The virion has a spherical or pleomorphic shape with 120 nm in diameter. This virus has an envelope decorated with large club-shaped spikes (S). Its genome is a linear positive-sense single-stranded ribonucleic acid (RNA) approximately 29.8–29.9 kilobases (kb) long^4^. SARS-CoV-2 sequence is closely related to bat SARS-like coronaviruses found in *Rhinolophus sinicus* (88% identity) and SARS-CoV found during 2002–2003 (79% identity)^5^. This virus is easily transmitted via inhalation. The incubation period is between 3 and 14 days (5 days on average)^6–8^. In the case of omicron, the incubation period is even shorter (2–4 days on average)^9–11^. Typical presenting symptoms are fever, cough, dyspnea, myalgia, and headache. Some patients may have sore throat, runny nose, diarrhea, nausea, and vomiting^12^. Most patients usually have mild and self-limiting illnesses. Nevertheless, severe pneumonia and death can develop in a portion of patients, especially the elderly and individuals with chronic illnesses^13^. A previous study found that SARS-CoV-2 was shed in the patient’s respiratory secretions as early as 4 days before the beginning of symptoms which could lead to pre-symptomatic transmission^14^. The patients are most highly contagious between 2 days before and 1 day after symptom onset^15^. So far, three antiviral drugs have received approval (remdesivir and nirmatrelvir/ritonavir) or EUA (molnupiravir) from The United States Food and Drug Administration (U.S. FDA) for the management of COVID-19. These drugs are recommended to treat a patient with mild-to-moderate illness at high risk of progression to severe disease. The antiviral therapies should be started as early as possible for the best outcome, within 5 days of symptom onset^16,17^. For a patient with severe illness, dexamethasone alone or in combination with one of the immunomodulators (baricitinib or tocilizumab) should be administered in conjunction with remdesivir^18^.

Because the symptoms and signs of COVID-19 are undistinguished from other acute respiratory tract infections, laboratory investigation is required to make a definite diagnosis. Real-time reverse transcription polymerase chain reaction (RT-PCR), using a patient’s respiratory tract specimen, is a standard method recommended for the diagnosis of COVID-19. However, a typical real-time RT-PCR requires 2–4 h to report the result. Real-time RT-PCR results revealed that SARS-CoV-2 RNA levels in respiratory samples were highest from the first to the seventh days of symptom onset^19–21^. Then the levels gradually decreased and became undetected in the third week of symptom onset. On average, patients with mild illness can have positive real-time RT-PCR results for up to 14 days after the onset of illness. Meanwhile, in severely ill patients, the results can be positive for up to 21 days after the onset of symptoms^22^. Although antigen test kit (ATK) can be used for screening COVID-19 within 15–30 min, the drawback is its limited sensitivity. Detection of SARS-CoV-2 antigen is most sensitive only during the period when a high level of virus is present in the patient’s respiratory secretion, i.e., between 1–3 days before and the first 5–7 days after the onset of symptoms^23^.

In recent years, rapid molecular assays have been developed for the detection of SARS-CoV-2 RNA. In addition to their high sensitivity, the results can be reported quickly within 15–30 min which is suitable for use as point-of-care testing (POCT). Rapid molecular assays may be divided into two groups based on their principles of nucleic acid amplification. The first group is based on RT-PCR, such as Cobas Liat SARS-CoV-2 (Roche, Switzerland)^24^, Accula SARS-CoV-2 (Mesa Biotech, USA)^25^, and Visby Medical COVID-19 (Visby Medical, USA)^26^ and the second group is based on isothermal amplification, such as ID Now COVID-19 (Abbott, USA)^27^, Cue COVID-19 (Cue Health, USA)^28^, and Lucira Check It COVID-19 (Lucira Health, USA)^29^.

**Fig. 1:**
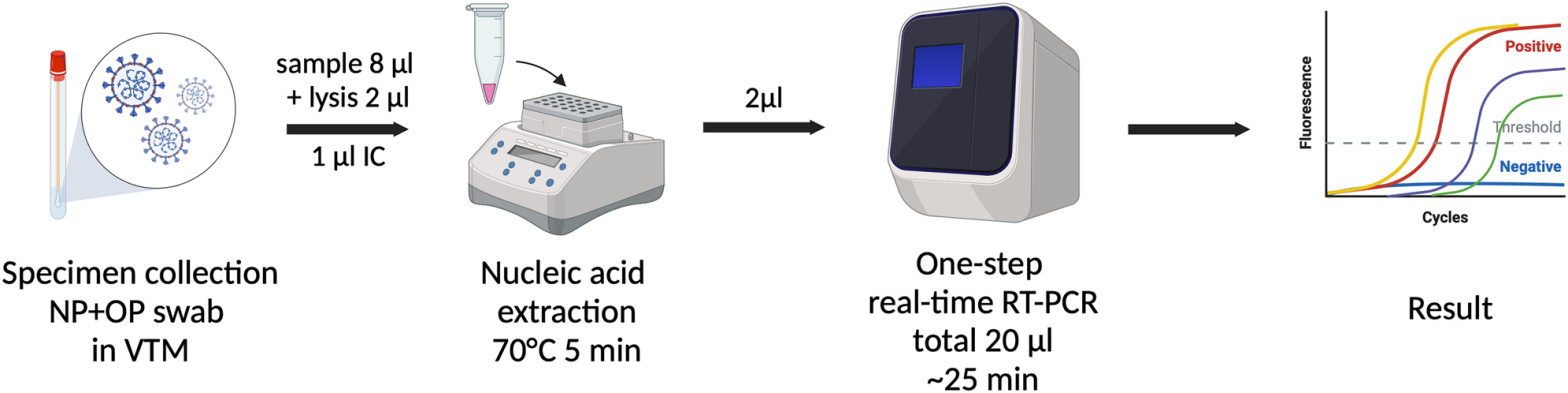
Workflow of SARS-CoV-2 detection by one-step quadruplex rapid real-time RT-PCR assay (ƩS COVID-19). VTM; Viral transport medium, IC; Internal control. The figure was created with BioRender.com.

Notwithstanding, these rapid molecular assays for SARS-CoV-2 detection usually require an operation on specially designed instruments. Moreover, most of them are low throughput, only one sample can be analyzed at a time which may not be appropriate for use in medical facilities with many patients. So, this study developed a new one-step quadruplex rapid real-time RT-PCR assay for SARS-CoV-2 detection called “Super Speed inSpector of COVID-19” or “ƩS COVID-19” operating on an opened platform (Fig. 1). The assay could provide the result within 25 min as well as high throughput capability. This newly developed rapid molecular assay, ƩS COVID-19 could be used as an alternative method for prompt diagnosis of COVID-19 which would aid not only an appropriate treatment for a patient but also infection control.

## Results

### Development of rapid real-time RT-PCR (ƩS COVID-19)

**Fig. 2:**
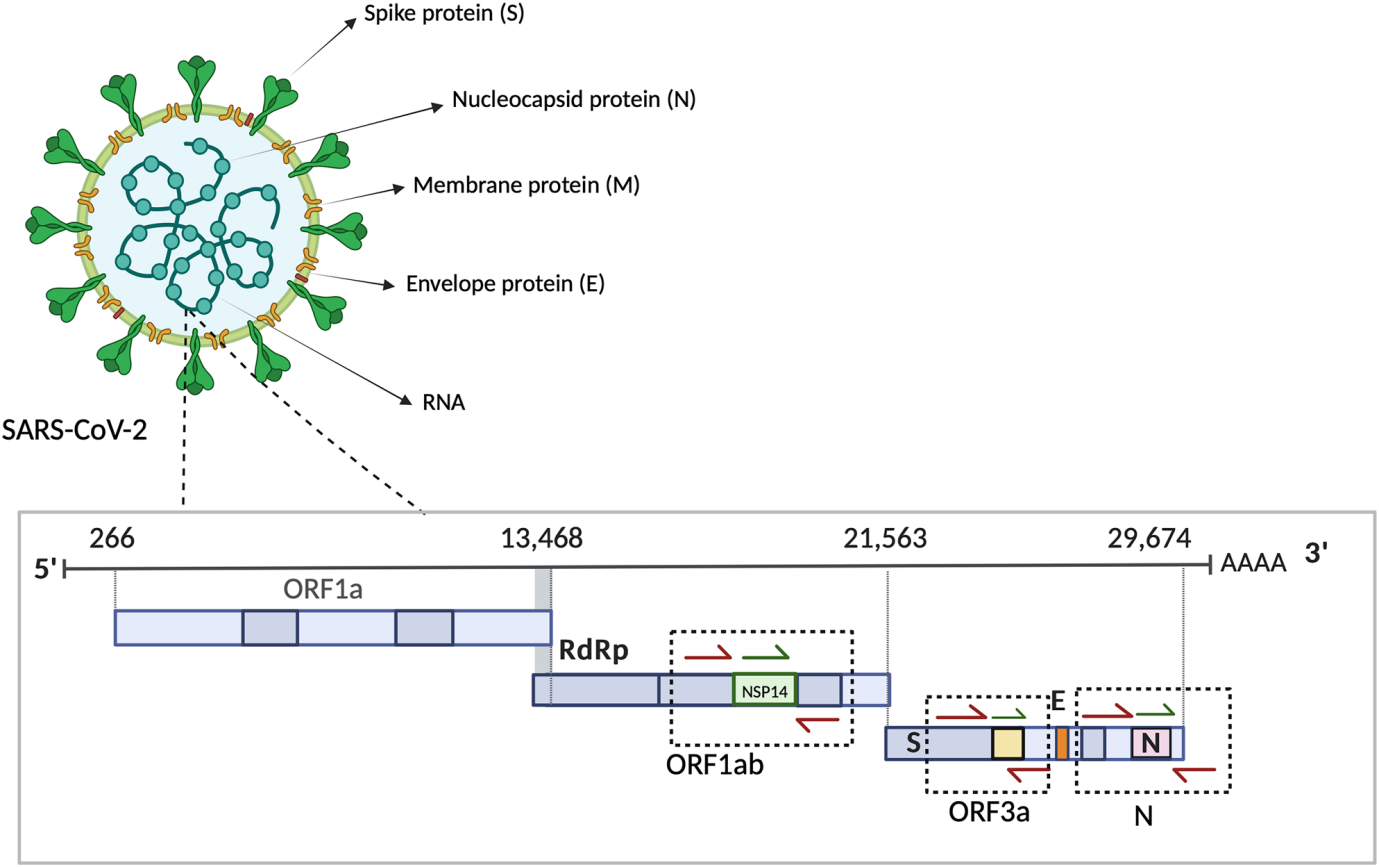
The SARS-CoV-2 genome was targeted by three sets of primers and probes specific for ORF1ab, ORF3a, and N genes. Red half arrows represented forward and reverse primers. Green half arrow represented probes. The figure was created with BioRender.com.

A newly developed one-step quadruplex real-time RT-PCR assay was designed to target ORF1ab, ORF3a, and N genes of SARS-CoV-2, and ASBVd as an internal control (Fig. 2). The initial setting used the standard protocol as described in the previous section. The total run time was 67 min 56 s.

**Fig. 3:**
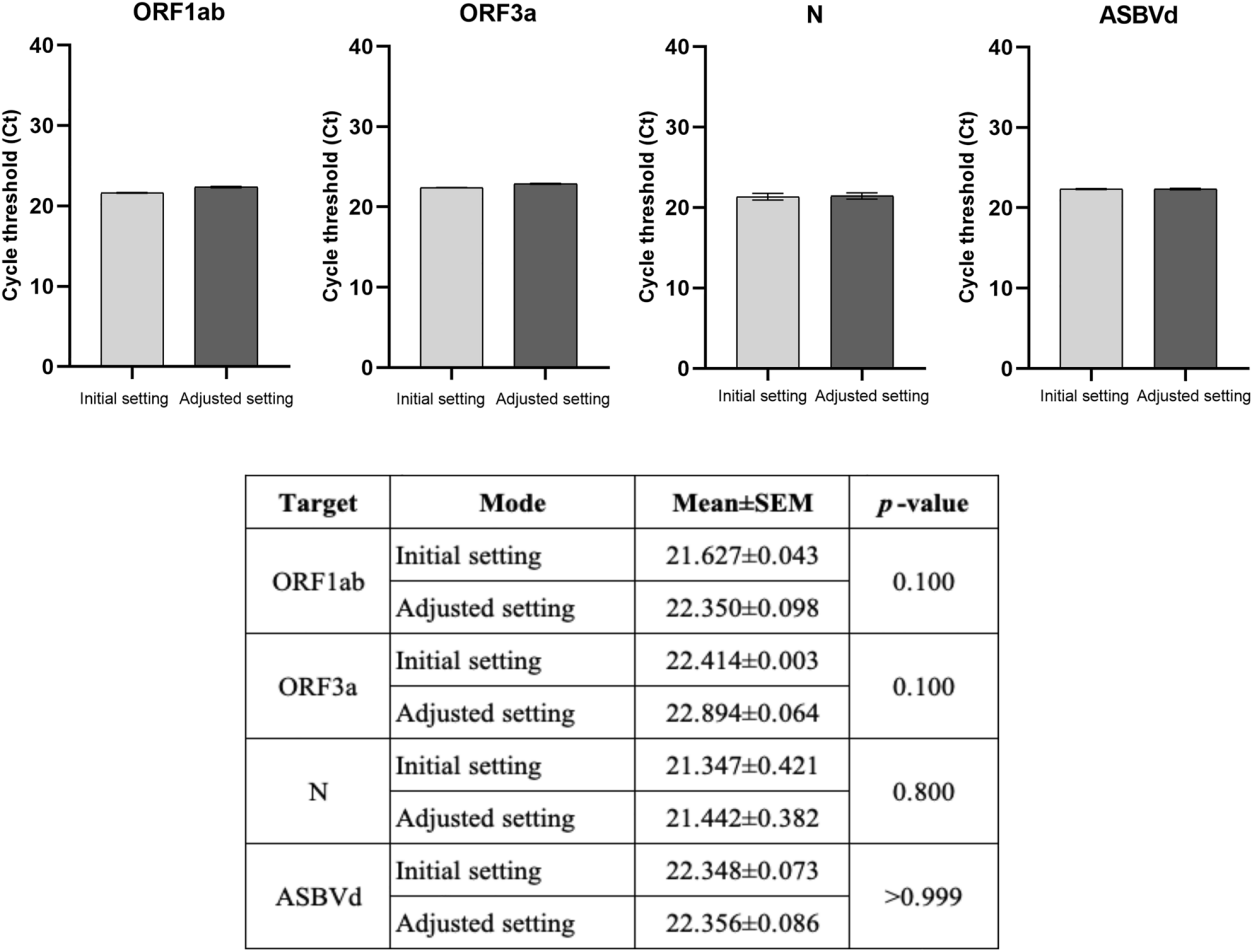
The performance of the real-time RT-PCR assay in the adjusted setting compared with the initial setting (the standard protocol). The mean ± SEM cycle threshold (Ct) values from an experiment with triplicate amplification were compared between two settings in each SARS-CoV-2 gene target (ORF1ab, ORF3a, and N genes) and the internal control (ASBVd). An unpaired *t-*test was used for comparison analysis. The significant difference was when *p* < 0.05.

To develop a rapid real-time RT-PCR assay, the protocol was first adjusted to a 5 min RT step followed by 1 min initial denaturation and 40 cycles of 2 s denaturation at 92 °C and 4 s annealing/extension at 60 °C. In addition, the fast mode of QuantStudio 5 which automatically adjusted the ramp rate from 1.6 °C/s in standard mode to 4.13 °C/s denaturation and 3.16 °C/s annealing was also applied. The performance of this adjusted setting was comparable with the initial setting as shown in Fig. 3.

Then, the assay was investigated step by step whether it could preserve its performance under extreme conditions. Hold time in each step including RT, initial denaturation, cycling denaturation, and annealing/extension was minimized.

**Fig. 4:**
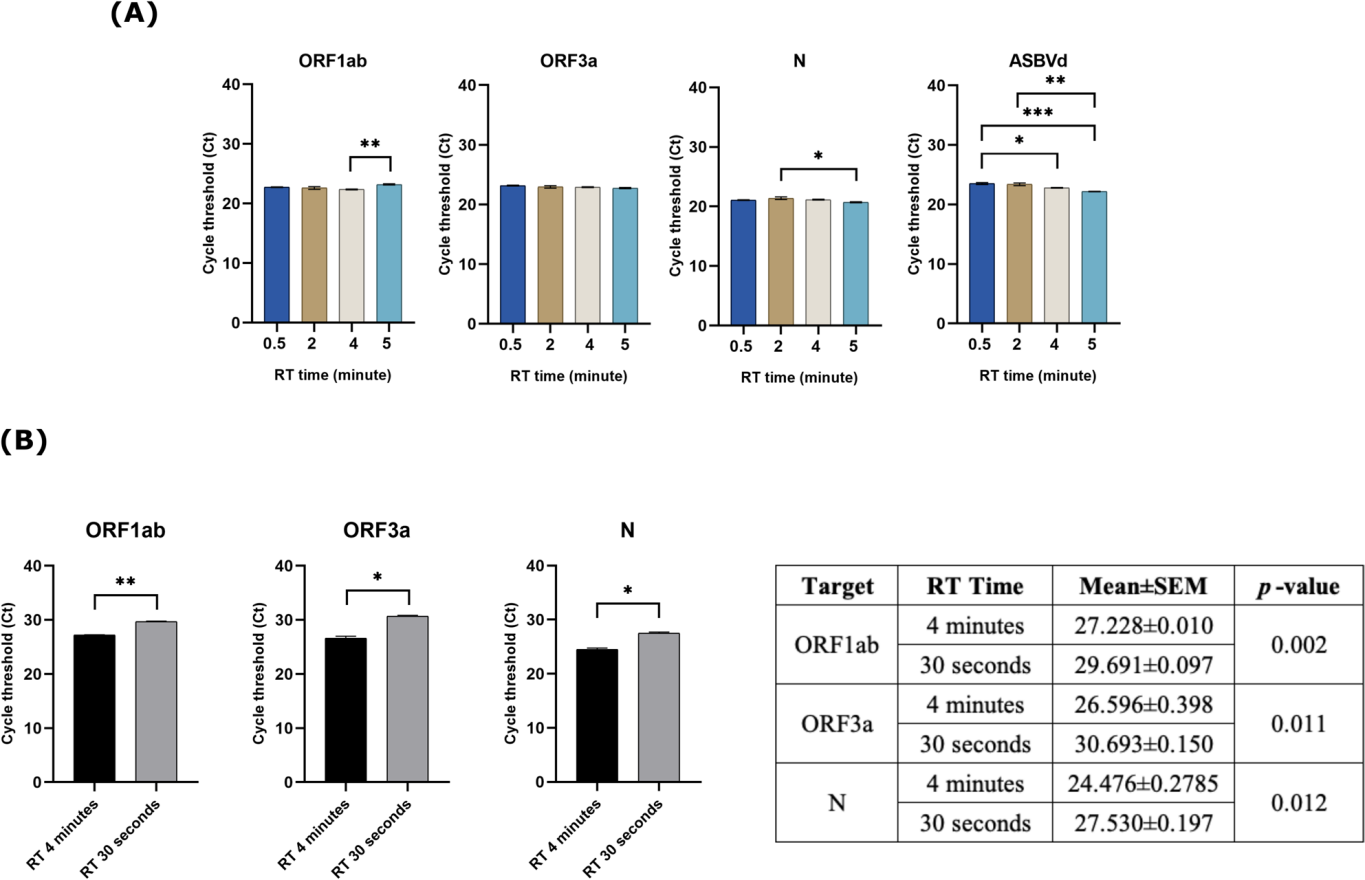
The performance of the adjusted real-time RT-PCR assay (**A**) The RT time was varied from 5 to 4 min, 2 min, and 30 s. The comparison of mean ± SEM Ct values of SARS-CoV-2 gene targets (ORF1ab, ORF3a, and N genes) and the internal control (ASBVd) was analyzed among the different RT time settings using the reference materials; (**B**) Comparing the mean ± SEM Ct values of SARS-CoV-2 gene targets in clinical samples at RT time between 4 min and 30 s. The significant level was calculated using an unpaired *t*-test and one-way ANOVA with Turkey’s multiple comparison test. The asterisk indicated the statistically significant difference. *p < 0.05, **p < 0.01, and ***p < 0.001.

Starting with RT, because the assay used WarmStart Luna Reverse Transcriptase (New England Biolabs, USA) which required incubation at 55 °C to ensure full activation, the idea of omitting the RT step by just letting the RT reaction occur while preparing the master mix at room temperature was not possible. Therefore, 5 min of RT time was compared with 4 min, 2 min, and 30 s using reference materials. Although the statistically significant difference in mean ± SEM Ct values was found, the mean difference was less than one cycle in ORF1ab (0.840) and N (0.756). Therefore, the reduction of RT time from 5 min down to 30 s very slightly affected the performance of the assay for all 3 SARS-CoV-2 gene targets. The most effect was found in ASBVd, the internal control, but the mean difference was still less than two cycles (Fig. 4A, Supplementary Table S2). Since the test was developed for use with the clinical specimen, five clinical specimens (two positive and three negative samples) were run instead of using the reference materials at RT time of 4 min and 30 s. The results revealed that 4 min was significantly better than 30 s for all 3 SARS-CoV-2 gene targets (Fig. 4B). As a result, the 4 min RT time was selected.

**Fig. 5:**
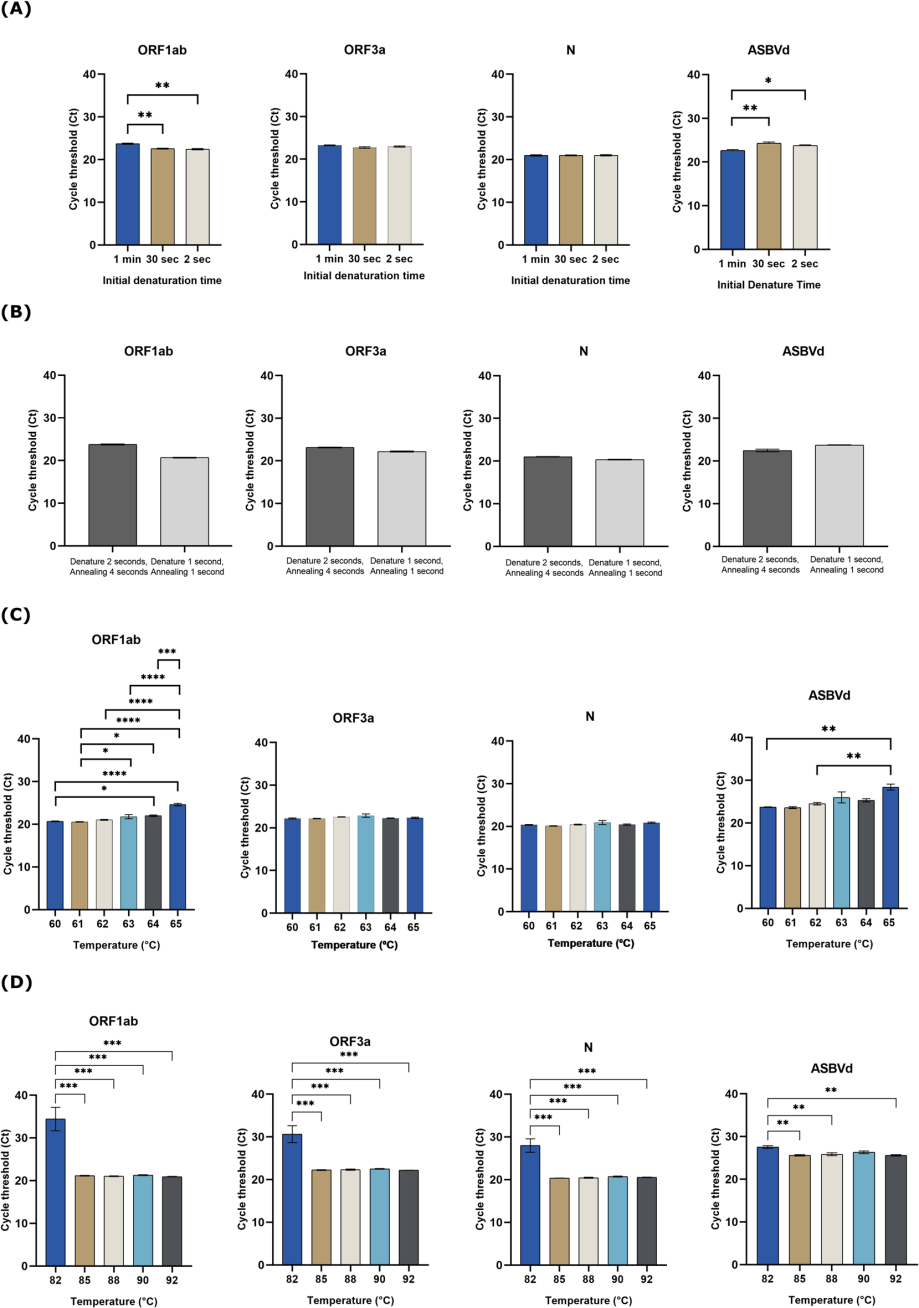
The performance of the real-time RT-PCR assay. (**A**) when varying initial denaturation time from 1 min to 30 and 2 s; (**B**) when cycling denaturation and annealing/extension time was compared between 2–4 s and 1–1 s; (**C**) when varying annealing/extension temperatures from 60 to 65 °C; (**D**) when varying denaturation temperatures from 82 to 85, 88, 90, and 92 °C. The comparison of mean ± SEM Ct values of SARS-CoV-2 gene targets (ORF1ab, ORF3a, and N genes) and the internal control (ASBVd) was analyzed among the different conditions. The significant difference was calculated using an unpaired *t*-test and one-way ANOVA with Turkey’s multiple comparison test. The asterisk indicated the statistically significant difference. *p < 0.05, **p < 0.01, ***p < 0.001, and ****p < 0.0001.

In addition to denaturing the target RNA template, an initial denaturation step was required for two other purposes. First, it allowed full activation of a Hot Start *Taq* DNA polymerase if used. Second, it inactivated a reverse transcriptase to avoid interference with subsequent PCR steps. Since the assay used an aptamer-based Hot Start *Taq* DNA polymerase (New England Biolabs, USA) swiftly becoming active once the incubation temperature was above 45 °C, we speculated that the hold time of this step might also be shortened. Thus, the initial denaturation time was varied from 1 min to 30 and 2 s. The results showed that the performance of the assay using a 2 s initial denaturation was not inferior to the default 1 min setting for all three SARS-CoV-2 gene targets. (Fig. 5A, Supplementary Table S3). These findings suggested that the full function of the Hot Start *Taq* DNA polymerase was gained without any interference between RT and PCR when such a short hold time was used. Thus, the 2 s initial denaturation time was chosen.

From previous knowledge native *Taq* DNA polymerase has the extension rate of 24 nucleotides per second at 55 °C and up to 45–60 nucleotides per second at 70–75°C^30,31^. To amplify the targets sized 76–105 bp, the annealing/extension time was supposed to be at least 2–3 s. However, considering the fact that most conventional real-time PCR instruments will spend a few seconds longer for plate read in each cycle, we hypothesized that amplification of such short products might be efficient with a 1 s annealing/extension time setting. Therefore, the cycling denaturation and annealing/extension times of 2 s and 4 s were compared with 1 s each. The results clearly showed that the performance of 1 s each was not significantly different from that of 2 s and 4 s (Fig. 5B, Supplementary Table S4). Therefore, 1 s denaturation and 1 s annealing/extension were selected.

Aside from incubation at various steps, real-time RT-PCR assay wastes substantial time ramping between denaturation and annealing/extension temperatures. Hence, the range between denaturation and annealing/extension temperatures should be reduced to shorten the run time. This could be achieved via two approaches, i.e., increment of the annealing/extension temperature and decrement of the denaturation temperature.

At first, the annealing/extension temperature was varied from 60 to 65 °C. The findings showed that the temperature could be raised to as high as 65 °C without detrimental effects on SARS-CoV-2 ORF3a and N gene targets. Meanwhile, only 60 and 61 °C worked best for the SARS-CoV-2 ORF1ab gene target and the internal control, without the delayed Ct (Fig. 5C, Supplementary Table S5).

Lastly, we predicted the melting temperature (Tm) of each amplicon using uMelt Quartz and found that the Tm of SARS-CoV-2 ORF1ab, ORF3a, and N amplicons were estimated as 84.5, 84, and 84 °C, respectively. At the same time, the predicted Tm of the ASBVd amplicon was 81.5 °C (Supplementary Fig. S1). We hypothesized that lowering the denaturation temperature down to 85 °C might be sufficient for an effective amplification. Thereby, the denaturation temperature was varied from 92 to 90, 88, 85, and 82 °C. The findings revealed that the denaturation temperature as low as 85 °C still gave an efficient amplification of all targets not different from the higher temperatures (Fig. 5D, Supplementary Fig. S2, Supplementary Table S6). Thus, denaturation at 85 °C was opted.

**Fig. 6:**
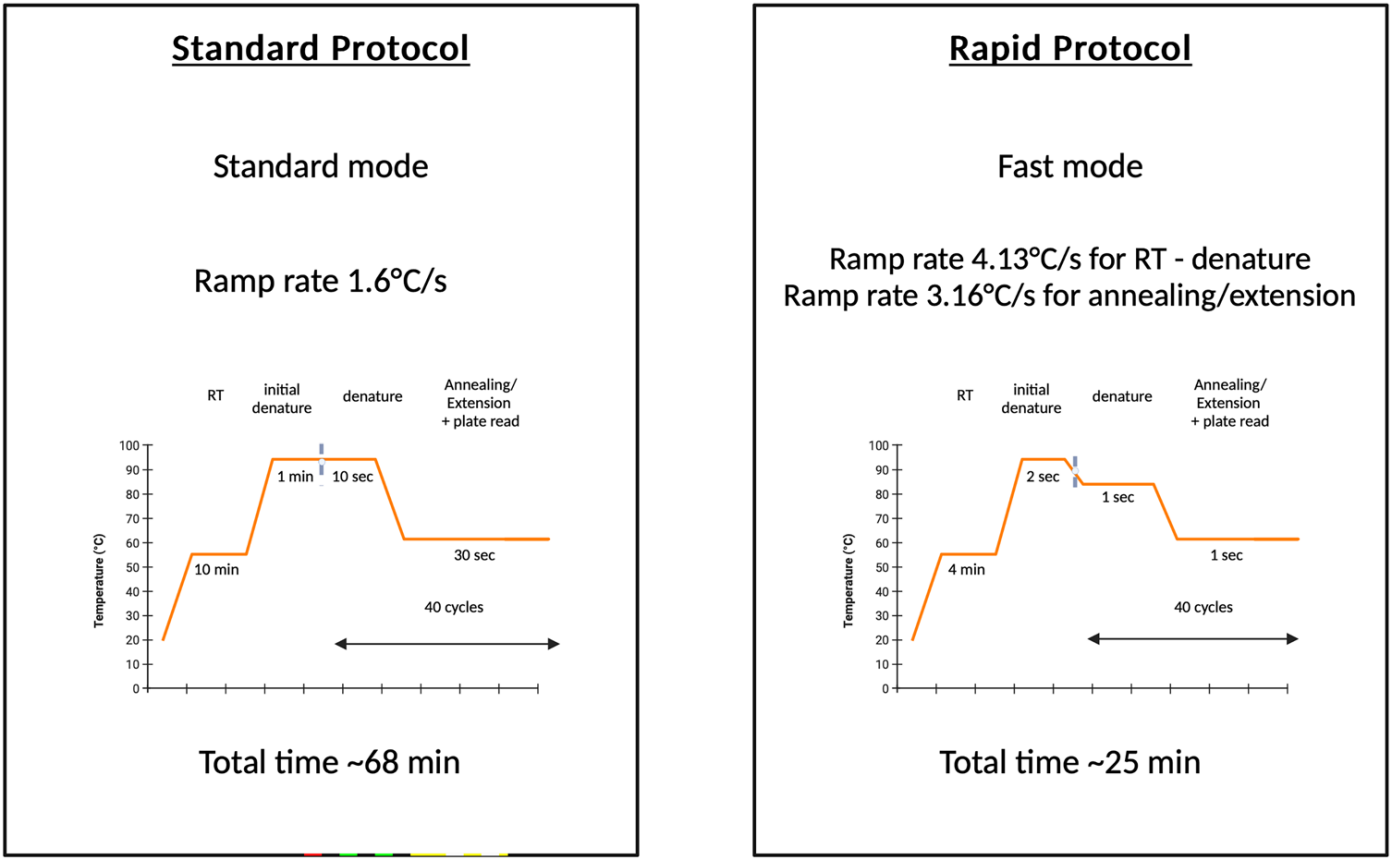
Time required by real-time RT-PCR (Standard protocol) and rapid real-time RT-PCR (ƩS COVID-19). The figure was created with BioRender.com.

Altogether, the extreme setting of our newly developed assay named “Super Speed inSpector of COVID-19” or “ƩS COVID-19” was a 4 min RT step at 55 °C followed by 2 s initial denaturation at 95 °C and 40 cycles of 1 s denaturation at 85 °C and 1 s annealing/extension at 60 °C. Total run time was reduced to 24 min 46 s (more than 60% reduction compared with the standard protocol) (Fig. 6).

### Sensitivity of ƩS COVID-19

**Fig. 7:**
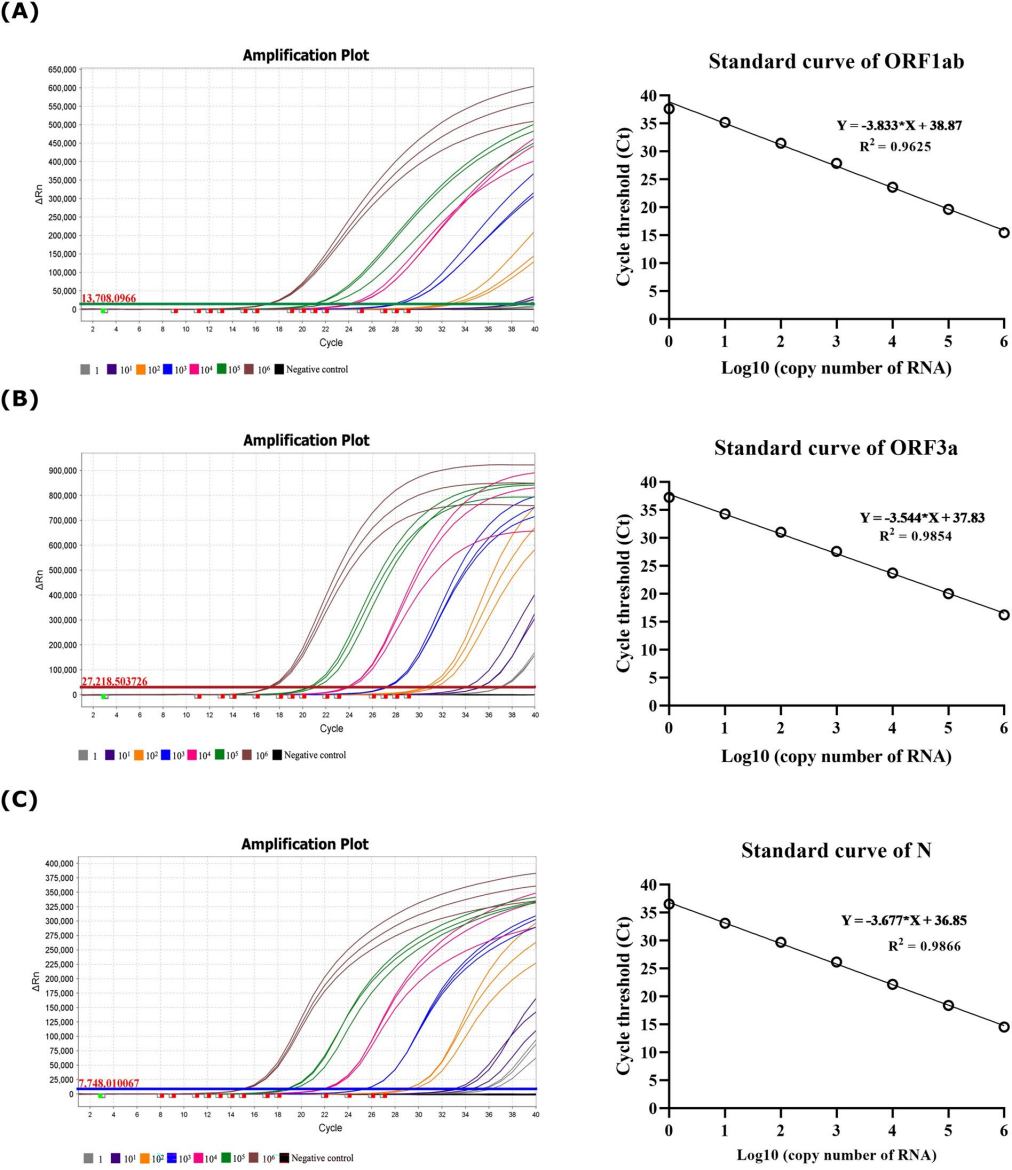
The sensitivity of the rapid real-time RT-PCR assay (ƩS COVID-19). The sensitivity of ƩS COVID-19 was determined using the reference SARS-CoV-2 RNA ranging from 10^6^ to 1 copies/reaction. Forty cycles of amplification were done in 20 replicates for each concentration of the reference RNA. The amplification plots and the standard curve of the (**A**) ORF1ab, (**B**) ORF3a, and (**C**) N genes of the SARS-CoV-2. The error bar represented the mean ± SEM.

Analytical sensitivity of ƩS COVID-19 was determined by testing the reference SARS-CoV-2 RNA which was tenfold serially diluted to achieve 10^6^ to 1 copies/reaction. Each concentration of the reference material was tested in 20 replicates. All agreements of the 20 replicated results were found 100% at all concentrations except at 1 copy/reaction was shown at 50% (10/20) for ORF1ab, 85% (17/20) for ORF3a, and 95% (19/20) for N. Hence, the limit of detection (LOD) from probit regression analysis of ORF1ab, ORF3a, and N genes were 1.835, 1.310, and 1 copy/reaction, respectively (Fig. 7, Supplementary Fig. S3).

### Specificity of ƩS COVID-19

**Fig. 8:**
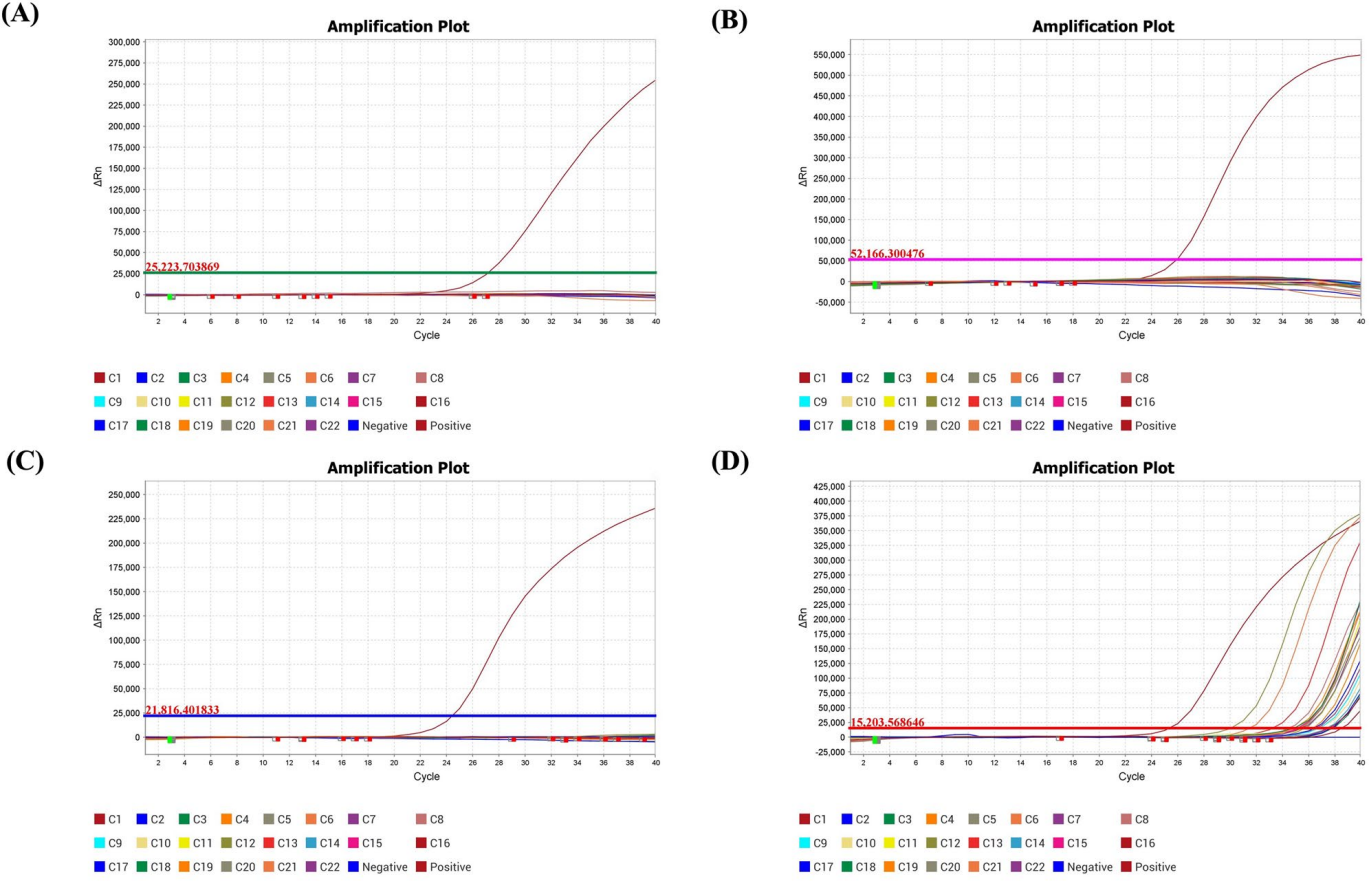
The specificity of the rapid real-time RT-PCR assay (ƩS COVID-19). The specificity of ƩS COVID-19 was determined using 22 respiratory samples positive for other 16 common respiratory viruses (C1-C22) detected by either BioFire Respiratory 2.1 Panel or QIAstat-Dx Respiratory SARS-CoV-2 Panel. The amplification plots of the (**A**) ORF1ab, (**B**) ORF3a, (**C**) N genes of SARS-CoV-2, and (**D**) the internal control (ASBVd).

To determine the specificity, 22 respiratory samples positive for other 16 common respiratory viruses detected using either BioFire Respiratory 2.1 Panel or QIAstat-Dx Respiratory SARS-CoV-2 Panel were tested with ƩS COVID-19. The aforementioned respiratory viruses included coronavirus OC43, coronavirus 229E, coronavirus NL63, coronavirus HKU1, influenza A(H1N1)pdm09 virus, influenza A(H3N2) virus, influenza B virus, respiratory syncytial virus, human metapneumovirus, parainfluenza virus type 1, parainfluenza virus type 2, parainfluenza virus type 3, parainfluenza virus type 4, rhinovirus/enterovirus, adenovirus, and human bocavirus. The results confirmed that the newly developed assay had no cross-reactivity with any of these common respiratory pathogens (Fig. 8).

### Validation of ƩS COVID-19 in clinical samples

The newly developed assay was further validated with 83 SARS-CoV-2 positive and 100 SARS-CoV-2 negative samples determined by FDA EUA approved Cobas SARS-CoV-2 system. The results showed that ƩS COVID-19 could detect SARS-CoV-2 RNA in 77 out of 83 samples. Six discordant samples were reported as inconclusive by ƩS COVID-19, i.e., only one target gene of SARS-CoV-2 was detected (N in four samples, ORF1ab in one sample, and ORF3a in one sample). However, all these six samples were confirmed as SARS-CoV-2 detected by another FDA EUA approved assay, the Cobas SARS-CoV-2 & Influenza A/B test for use on the Cobas Liat System (Roche, Switzerland). Meanwhile, ƩS COVID-19 did not detect SARS-CoV-2 RNA in any negative samples. Therefore, the diagnostic sensitivity, specificity, positive predictive value (PPV), negative predictive value (NPV), and accuracy of ƩS COVID-19 were 92.8%, 100%, 100%, 97.1%, and 96.7% respectively. Almost perfected agreement (Cohen’s Kappa 0.93) was found between both assays (Table 1).

**Table 1:** SARS-CoV-2 detection by the rapid real-time RT-PCR (ƩS COVID-19) compared with cobas^®^ SARS-CoV-2 for use on the cobas^®^ 6800 system.

ƩS COVID-19 results	cobas SARS-CoV-2 results		
	Detected	Undetected	Total
Detected	77	0	77
Undetected	6*	100	106
Total	83	100	183
Diagnostic sensitivity	92.8%		
Diagnostic specificity	100%		
Positive predictive value	100%		
Negative predictive value	97.1%		
Accuracy	96.7%		
Kappa	0.93		

*Six samples interpreted as inconclusive by ƩS COVID-19 were counted as undetected.

## Discussion

Accurate diagnosis of COVID-19 is crucial for patient management as well as infection control measures. Real-time RT-PCR is a standard method recommended for the definite diagnosis of COVID-19. Nevertheless, at least 2–4 h are required before obtaining the result which is not suitable for medical service in outpatient and emergency departments. In recent years, several rapid real-time RT-PCR assays for SARS-CoV-2 detection were developed in response to COVID-19 pandemic. These assays could be completed within 20–30 min while maintaining the sensitivity as high as conventional real-time RT-PCR. Most of these assays required an operation on a specially designed microfluidic device which allowed the reaction mixture to immediately move between the denaturation and annealing/extension chambers. For instance, Cobas Liat System (Roche, Switzerland) and PicoGene PCR1100 (Nippon Sheet Glass, Japan) could generate the results within 20 min by this approach^24,32,33^ while Q-POC (QuantuMDx Group, UK) could do the same in approximately 30 min^34,35^. However, most of these platforms could process just one clinical specimen per run which might be a bottleneck for medical services during an epidemic season. Therefore, this study developed a one-step quadruplex rapid real-time RT-PCR assay for SARS-CoV-2 detection named “ƩS COVID-19”, operating on an opened conventional real-time RT-PCR instrument. In addition to its rapid run time of less than 25 min, the newly developed assay could expand the throughput to as high as 94 samples per run.

The basis underlying the development of the rapid real-time RT-PCR assay comprised 4 main steps. First, primers with the highest Tm (60–65 °C or over) and short amplicons (approximately 70–100 bp) were designed. Usage of primers with high Tm would allow the adoption of two-step PCR instead of three-step PCR which would greatly reduce total run time. At the same time, short amplicons would aid in minimizing hold time in the next step. Second, a fast ramp rate was applied. In the standard mode of QuantStudio 5 used in this study, the ramp rate was constant at 1.6 °C/s. When the fast mode was opted, the ramp rates were automatically adjusted to 4.13 °C/s in the denaturation step and 3.16 °C/s in the annealing step. For some real-time PCR instruments that do not have fast mode, e.g., CFX96 real-time PCR detection system (Bio-Rad Laboratories, USA), fast ramp rate can also be applied by manually changing the ramp rate setting for each step to the maximum (5 °C/s). Third, hold time in each step (RT, initial denaturation, cycling denaturation, and annealing/extension) was minimized. In this study, although RT time could be decreased to as short as 30 s for the reference materials, a 4 min RT time was required when testing with the clinical specimens. This discrepancy was also observed by a previous study showing that a 30 s RT time was sufficient for pure SARS-CoV-2 RNA but at least 5 min was needed for extraction-free clinical specimens. This might be affected by the presence of inhibitors in the patient’s respiratory samples^36^. In this study, the amplification of short amplicons (ranging from 76 to 105 bp) using the ultra-short hold time (1 s denaturation and 1 s annealing/extension time) and a Hot Start *Taq* DNA Polymerase was successfully implemented. Several previous studies also reported the success of using this ultra-short setting in the development of rapid real-time RT-PCR assays for animal viruses^37,38^ and SARS-CoV-2^39,40^. Lastly, the range between denaturation and annealing/extension temperatures was narrowed. This could be done by lowering the denaturation temperature and/or raising the annealing/extension temperature. To our knowledge, this was the first study that introduced the approach of predicting the Tm of the amplicons and determining the possible lowest denaturation temperature to be used in the assay. By this approach, lowering the denaturation temperature down to 85 °C which was much lower than typical PCR (94–96 °C) was enabled.

Upon applying all the aforementioned approaches, the assay’s run time could be shortened to less than 25 min which was more than 60% reduction compared with the standard real-time RT-PCR protocol. The speed of ƩS COVID-19 was close to other rapid real-time RT-PCR assays for SARS-CoV-2 detection. In a previous study, Lownik JC et al. reported the development of a rapid monoplex real-time RT-PCR assay for SARS-CoV-2 detection using CDC N1 primers and probe. Their assay relied on 5 min RT time and 10 s annealing/extension time. Nonetheless, operating on a capillary-based LightCycler 1.5 instrument (Roche, Switzerland) which had a much faster ramp rate (20 °C/s) allowed the assay to be completed in 20 min^36^. In another study, Milosevic J et al. reported the development of three parallel rapid real-time RT-PCR assays for SARS-CoV-2 detection using CDC N1, N2, and human RNase P (RP) primers and probes. Although their assays also depended on 40 cycles of 1 s denaturation and 1 s annealing/extension time, the denaturation temperature was set at 95 °C. These assays were operated on CFX96 real-time PCR detection system and could be completed in 30 min, a bit longer than ours^40^. In another study, Bustin S et al. reported the development of a multiplex rapid RT-PCR called CoV2-ID targeting 3 SARS-CoV-2 genes (NSP10, NSP12, and N). The assay also carried on 40 cycles of 1 s denaturation and 1 s annealing/extension steps, at 95 °C and 60 °C, respectively. Nevertheless, they used multiple cycle fluorescence detection (MCFD) which detected signal at only cycles 8 (for baseline), 15, 20, 25, 30, and 35 instead of real-time detection. In this way, the CoV2-ID could be completed in less than 20 min^39^. Since SARS-CoV-2 mutated continuously, three conserved SARS-CoV-2 gene targets were included in the newly developed assay to ensure that the performance of the assay was not affected if one of these gene targets mutated. In the previous study, CoV2-ID also included 3 SARS-CoV-2 gene targets (NSP10, NSP12, and N). Nonetheless, the NSP12 target was intentionally added in order to improve the sensitivity of the assay and was detected in the same channel as the NSP10 target^39^.

The newly developed assay could detect SARS-CoV-2 ORF1ab, ORF3a, and N gene targets as low as 1.835, 1.310, and 1 copy/reaction, respectively. This assay had high diagnostic sensitivity (92.8%), specificity (100%), and accuracy (96.7%) when compared with FDA EUA approved Cobas SARS-CoV-2 System. The performance of ƩS COVID-19 was comparable to rapid real-time RT-PCR assays developed by other researcher groups. The extraction-free LightCycler SARS-CoV-2 assay using CDC N1 primers and probe was reported to reach the LOD of 3 copies/reaction. It had a positive percent agreement (PPA) of 97.6% and a negative percent agreement (NPA) of 100% when compared with either the University of Washington SARS-CoV-2 real-time RT-PCR or Aptima SARS-CoV-2 assays (Hologic, USA)^36^. Three parallel CFX96 SARS-CoV-2 assays using CDC N1, N2, and RP primers and probes were reported to yield the LOD of 25 copies/reaction for both N1 and N2 targets. They had a PPA of 100%, NPA of 100%, and overall percent agreement (OPA) of 100% when compared with Xpert Xpress SARS-CoV-2 (Cepheid, USA)^40^. The CoV2-ID was reported to have the LOD of 2 and 5 copies/reaction for NSP10 and NSP12, respectively. The assay had a sensitivity of 100%, specificity of 100%, and accuracy of 100% when compared with the VIASURE SARS-CoV-2 real-time PCR detection kit (Certest Biotec, Spain)^39^.

In addition, this was the first study that described the use of a viroid RNA as an internal control for human virus testing. Single-stranded viroid RNA is extensively self-complementary forming a robust secondary structure^41,42^. Its secondary structure seemed to provide more durability and resistance to RNases than host-derived RNA^43^. In addition to its native difficult template, this study designed the internal control amplicon size larger than the target amplicons and successfully utilized it as the internal control for the non-competitive rapid real-time RT-PCR. In a previous study, Botermans M et al. also reported the usage of a viroid as an internal control in multiplex real-time RT-PCR for the detection of other viroids causing diseases in plants^43^. In another study, Dinkle KE et al. reported the use of hepatitis D virus (HDV) RNA, which also had the rod-like secondary structure, as an internal control for nested multiplex RT-PCR of other respiratory viruses^44^.

Apart from switching to using a real-time PCR instrument with a faster ramp rate, there is still room to improve the performance and versatility of our rapid real-time RT-PCR assay. The former; amplification of longer amplicons might be enabled if an alternative high-speed DNA polymerase, such as KAPA2G Fast, Klentaq1, and KOD polymerases, was used^45–47^. The latter, hold time might be further shortened by usage of a higher concentration of primers (5–20 μM) in a smaller reaction volume (5–10 μl)^36,45,48^. However, these approaches could come at the expense of a higher cost.

In conclusion, based on strategies including designing primers with high Tm and short amplicons, applying fast ramp rate, minimizing hold time, and reducing the range between denaturation and annealing/extension temperatures; we could develop a one-step quadruplex rapid real-time RT-PCR assay for SARS-CoV-2 detection that could be accomplished within 25 min. The newly developed rapid real-time RT-PCR assay was highly sensitive, specific, and fast suitable for use as an alternative method to support laboratory diagnosis of COVID-19 in outpatient and emergency departments.

## Materials and methods

### Primer and probe design

One hundred and fourteen SARS-CoV-2 nucleotide sequences were retrieved from the GenBank and Global Initiative on Sharing Avian Influenza Data (GISAID) databases (their accession numbers were listed in Supplementary Table S1). Three pairs of primers and three hydrolysis probes targeting the conserved regions of ORF1ab, ORF3a, and N genes of SARS-CoV-2 were designed using the Primer-Blast program (Fig. 2)^49^. A pair of primers and a hydrolysis probe specific to Avocado sunblotch viroid (ASBVd), which was used as an exogenous internal control, were designed using the PrimerQuest program (Integrated DNA Technologies, USA) available from https://www.idtdna.com/SciTools. All primers and probes were synthesized by Macrogen (South Korea). The sequences of the primers and probes were shown in Table 2. The melting temperature of each amplicon was predicted using the uMELT Quartz program^50^.

**Table 2:** The primers and probes used in the rapid real-time RT-PCR assay (ƩS COVID-19).

Names	Sequences (5ʹ–3ʹ)	Amplicon size (bp)
Wuh_ORF1ab_F	CTGAGCGCACCTGTTGTCTA	76
Wuh_ORF1ab_R	TGCCAACAGGCATAAGTGTC	
Wuh_ORF1ab_P	HEX-ACGTGCCACATGCTTTTCCACTGCT-BHQ1	
Wuh_ORF3a_F	AAGCAAGGTGAAATCAAGGATGC	80
Wuh_ORF3a_R	GGGAGTGAGGCTTGTATCGG	
Wuh_ORF3a_P	FAM-AGATTTTGTTCGCGCTACTGCAACGA-BHQ1	
Wuh_N_F	GCAGACGTGGTCCAGAACAA	92
Wuh_N_R	TGCAATTTGCGGCCAATGTT	
Wuh_N_P	Cy5-ACCCAAGGAAATTTTGGGGACCAGGA-BHQ2	
ASBVd_F	AGGAGGAGTCGTGGTGAACT	105
ASBVd_R	GACTCATCAGTGTTCTTCCCATCTT	
ASBVd_P	ROX-TCTCTTGATCACTTCGTCTCTTCAGGGA-BHQ2	

*F* forward, *R* reverse, *P* probe.

### Reference materials

Three RNA transcripts of SARS-CoV-2 ORF1ab, ORF3a, and N genes were transcribed in vitro by HiScribe T7 quick high yield RNA synthesis kit (New England Biolabs, USA) using synthetic DNA fragments (based on SARS-CoV-2 reference genome, isolate Wuhan-Hu-1, GenBank accession number NC_045512.2) appended with T7 promoter sequence (Macrogen, South Korea) as their templates. One microgram of the template DNA was mixed with NTP Buffer Mix (6.7 mM each) and 2 μl of T7 RNA Polymerase Mix. Nuclease-free water was added to achieve a total reaction volume of 30 μl. The reaction mixture was incubated for 16 h. The template DNA was subsequently removed by adding 30 μl of nuclease-free water and 2 μl of DNase I (RNase-free), followed by incubation at 37 °C for 15 min. Then the transcribed RNA was purified using monarch RNA cleanup kit (50 µg) (New England Biolabs, USA) according to the manufacturer’s recommendation. The concentration of the purified RNA was quantified by Qubit RNA BR assay kit (Thermo Fisher Scientific, USA) following the protocol recommended by the manufacturer. Genome equivalents per microliter were calculated using an RNA copy number calculator^51,52^. These SARS-CoV-2 transcripts were used as reference materials in subsequent experiments. ASBVd RNA (based on the reference sequence, GenBank accession number NC_001410.1) was also transcribed by the same method and was used as an exogenous internal control.

### Clinical samples

A total of 205 leftover combined nasopharyngeal and oropharyngeal swabs in viral transport medium (VTM) were obtained from the virology laboratory, microbiology department, King Chulalongkorn Memorial Hospital, Thai Red Cross, Bangkok, Thailand, with permission from the Director of Chulalongkorn Memorial Hospital in accordance with IRB regulations, without the patient’s consent, which is a prerequisite for IRB approval. Only unidentified leftover specimens were utilized in our experiment. There was no direct interaction with the patient and no collection of information or retrieval of data related to the patient. Thus, no patient’s consent was required and informed consent has been waived by the Institutional Review Board of the Faculty of Medicine, Chulalongkorn University. These samples were from suspected COVID-19 patients at King Chulalongkorn Memorial Hospital, Bangkok, Thailand from July 17th, 2021, to August 19th, 2022. All of them were prior routinely detected for SARS-CoV-2-RNA. There were 83 SARS-CoV-2 positive and 100 SARS-CoV-2 negative samples determined by Cobas SARS-CoV-2 on the Cobas 6800 System (Roche, Switzerland); and 22 samples positive for other common respiratory viruses detected by using either BioFire Respiratory 2.1 Panel (bioMérieux, France) or QIAstat-Dx Respiratory SARS-CoV-2 Panel (Qiagen, Germany). The leftover samples were stored anonymously at − 80 °C after routine testing.

### Nucleic acid extraction

The SARS-CoV-2 RNA was extracted from the combined nasopharyngeal and oropharyngeal swabs in VTM using heating unextracted diagnostic samples to obliterate nucleases (HUDSON) protocol with modification^53^. In brief, 8 μl of the clinical sample was mixed with 2 μl of lysis buffer containing 5 mM tris(2-carboxyethyl)phosphine (TCEP) (Thermo Fisher Scientific, USA) and 0.1 mM ethylenediaminetetraacetic acid (EDTA) (Bio Basic, Canada). Then, 1 μl of the internal control (ASBVd RNA) was added, followed by heat inactivation at 70 °C for 5 min. The extracted RNA was stored at − 80 °C until further use.

### Real-time RT-PCR (standard protocol)

The multiplex real-time RT-PCR was performed using Luna Universal Probe One-Step RT-qPCR Kit (New England Biolabs, USA). Twenty microliters of a reaction volume contained 1× Luna Universal Probe One-Step Reaction Mix, 1× Luna WarmStart RT Enzyme Mix, 0.4 μM each of Wuh_ORF1ab_F, Wuh_ORF1ab_R, Wuh_ORF3a_F, Wuh_ORF3a_R, Wuh_N_F, Wuh_N_R, ASBVd-F, and ASBVd-R, and 0.2 μM each of Wuh_ORF1ab_P, Wuh_ORF3a_P, Wuh_N_P, and ASBVd-P, and 2 μl of the extracted RNA. The amplification reaction was performed on QuantStudio 5 real-time PCR system (Thermo Fisher Scientific, USA). The thermocycling protocol was as follows: RT at 55 °C for 10 min, initial denaturation at 95 °C for 1 min, followed by 40 cycles of denaturation at 95 °C for 10 s and annealing/extension at 60 °C for 30 s. A plate read was included at the end of each annealing/extension step. An external negative control was also included in each run of the test using nuclease-free water in substitute of the extracted RNA.

### Rapid real-time RT-PCR (ƩS COVID-19)

The rapid real-time RT-PCR (ƩS COVID-19) was performed using the same procedure as the previous section but with a modified thermocycling protocol. The RT-PCR cycle steps were programmed as follows: RT at 55 °C for 4 min, initial denaturation at 95 °C for 2 s, followed by 40 cycles of denaturation at 85 °C for 1 s and annealing/extension at 60 °C for 1 s. The fluorescence signal was collected at the end of each annealing/extension step. The result was interpreted as SARS-CoV-2 detected when at least two out of three SARS-CoV-2 gene targets were detected with Ct less than 40. The result was interpreted as SARS-CoV-2 undetected if none of all 3 SARS-CoV-2 gene targets were detected and the internal control was detected with Ct less than 40. If only one out of three SARS-CoV-2 gene targets was detected with Ct less than 40, the result was interpreted as inconclusive.

### Statistical analysis

GraphPad Prism version 10 for Windows (Dotmatics, USA) was used to analyze the data. The mean ± standard error of measurement (SEM) of the Ct value was calculated. Mean Ct values of the different real-time RT-PCR protocols were compared using the unpaired* t*-test and one-way ANOVA with Turkey’s multiple comparison test. To obtain the significant difference, *p*-value < 0.05 was used. Linear regression analysis between the Ct value and log copy number of the reference materials was performed. To calculate the limit of detection (LOD) at the 95% probability level, probit regression analysis was performed using MedCalc version 22.032 (MedCalc Software, Belgium). By using the FDA EUA approved Cobas SARS-CoV-2 for use on the Cobas 6800 System as a reference method; the diagnostic sensitivity, specificity, positive predictive value, negative predictive value, and accuracy of ƩS COVID-19 were calculated. Cohen’s Kappa coefficient was computed to measure the agreement between two methods.

## Supplementary Information

Supplementary Information.

## Data Availability

The data supporting the result and the nucleotide sequences from GenBank and Global Initiative on Sharing Avian Influenza Data (GISAID) databases for designing primers and probes in this study were shown in the Supplementary file.
