# Supplementary material for: ƩS COVID-19 is a rapid high throughput and sensitive one-step quadruplex real-time RT-PCR assay
Source: Sci Rep. 2024 Sep 4;14:20590. doi: 10.1038/s41598-024-71705-8 (PMC11374890; doi:10.1038/s41598-024-71705-8)
Supplement: Supplementary file 1 — Supplementary Information. [file 41598_2024_71705_MOESM1_ESM.pdf]

# ΣS COVID-19 is a rapid high throughput and sensitive one-step quadruplex real-time RT-PCR assay

Ekasit Kowitdamrong[a,b*], Sasiprapa Anoma[a,b], Thitiya Loykaew[c], Pokrath Hansasuta[a], Parvapan Bhattarakosol[a,b]

[a]Department of Microbiology, Faculty of Medicine, Chulalongkorn University, Bangkok 10330, Thailand.

[b]Center of Excellence in Applied Medical Virology, Chulalongkorn University, Bangkok 10330, Thailand.

[c] Department of Microbiology, King Chulalongkorn Memorial Hospital, Thai Red Cross, Bangkok 10330, Thailand.

*Corresponding author

Email: ekasit.k@chula.ac.th

**Supplementary Table S1.** Accession numbers of SARS-CoV-2 nucleotide sequences from GenBank and Global Initiative on Sharing Avian Influenza Data (GISAID) databases used for designing primers and probes in this study.

| Sequence | Accession number | Source |
|---|---|---|
| 1 | NC_045512.2 | GenBank |
| 2 | EPI ISL 402124 | GISAID |
| 3 | MW059036.1 | GenBank |
| 4 | LC547526.1 | GenBank |
| 5 | MT358639.1 | GenBank |
| 6 | MT451147.1 | GenBank |
| 7 | MT470103.1 | GenBank |
| 8 | MT461606.1 | GenBank |
| 9 | MT292570.1 | GenBank |
| 10 | MT077125.1 | GenBank |
| 11 | MT350282.1 | GenBank |
| 12 | MT447155.1 | GenBank |
| 13 | MT447176.1 | GenBank |
| 14 | MW079429.1 | GenBank |
| 15 | MT907520.2 | GenBank |
| 16 | MT919525.1 | GenBank |
| 17 | MT919526.1 | GenBank |
| 18 | EPI_ISL_493182 | GISAID |
| 19 | EPI_ISL_529217 | GISAID |
| 20 | EPI_ISL_529213 | GISAID |
| 21 | EPI_ISL_539548 | GISAID |
| 22 | EPI_ISL_466615 | GISAID |

| | | |
|---|---|---|
| 23 | EPI_ISL_418345 | GISAID |
| 24 | EPI_ISL_406862 | GISAID |
| 25 | EPI_ISL_412974 | GISAID |
| 26 | EPI_ISL_403932 | GISAID |
| 27 | EPI_ISL_447025 | GISAID |
| 28 | EPI_ISL_437618 | GISAID |
| 29 | EPI_ISL_455591 | GISAID |
| 30 | EPI_ISL_512861 | GISAID |
| 31 | EPI_ISL_708823 | GISAID |
| 32 | EPI_ISL_708807 | GISAID |
| 33 | EPI_ISL_768538 | GISAID |
| 34 | EPI_ISL_896591 | GISAID |
| 35 | EPI_ISL_833041 | GISAID |
| 36 | EPI_ISL_777011 | GISAID |
| 37 | EPI_ISL_777016 | GISAID |
| 38 | MW494424.1 | GenBank |
| 39 | EPI_ISL_877766 | GISAID |
| 40 | EPI_ISL_660190 | GISAID |
| 41 | EPI_ISL_660567 | GISAID |
| 42 | EPI_ISL_1491581 | GISAID |
| 43 | EPI_ISL_3797062 | GISAID |
| 44 | EPI_ISL_5659409 | GISAID |
| 45 | EPI_ISL_1495989 | GISAID |
| 46 | EPI_ISL_3534190 | GISAID |
| 47 | EPI_ISL_3534194 | GISAID |
| 48 | EPI_ISL_3534200 | GISAID |

| | | |
|---|---|---|
| 49 | EPI_ISL_5416129 | GISAID |
| 50 | EPI_ISL_5722891 | GISAID |
| 51 | EPI_ISL_1538429 | GISAID |
| 52 | EPI_ISL_3342067 | GISAID |
| 53 | EPI_ISL_3403350 | GISAID |
| 54 | EPI_ISL_3403352 | GISAID |
| 55 | EPI_ISL_3342068 | GISAID |
| 56 | EPI_ISL_3403354 | GISAID |
| 57 | EPI_ISL_3403355 | GISAID |
| 58 | EPI_ISL_5879609 | GISAID |
| 59 | EPI_ISL_2840595 | GISAID |
| 60 | EPI_ISL_2877029 | GISAID |
| 61 | EPI_ISL_3402017 | GISAID |
| 62 | EPI_ISL_792680 | GISAID |
| 63 | EPI_ISL_833167 | GISAID |
| 64 | EPI_ISL_1708575 | GISAID |
| 65 | EPI_ISL_3761614 | GISAID |
| 66 | EPI_ISL_2467219 | GISAID |
| 67 | EPI_ISL_7673858 | GISAID |
| 68 | EPI_ISL_2154650 | GISAID |
| 69 | EPI_ISL_2155435 | GISAID |
| 70 | EPI_ISL_2189311 | GISAID |
| 71 | EPI_ISL_1543981 | GISAID |
| 72 | EPI_ISL_6134092 | GISAID |
| 73 | EPI_ISL_3403348 | GISAID |
| 74 | EPI_ISL_3342064 | GISAID |

| | | |
|---|---|---|
| 75 | EPI_ISL_7379689 | GISAID |
| 76 | EPI_ISL_3342065 | GISAID |
| 77 | EPI_ISL_3403346 | GISAID |
| 78 | EPI_ISL_3342062 | GISAID |
| 79 | EPI_ISL_2877037 | GISAID |
| 80 | EPI_ISL_7649948 | GISAID |
| 81 | EPI_ISL_2877033 | GISAID |
| 82 | EPI_ISL_1111335 | GISAID |
| 83 | EPI_ISL_1477056 | GISAID |
| 84 | EPI_ISL_2921045 | GISAID |
| 85 | EPI_ISL_3464547 | GISAID |
| 86 | EPI_ISL_6590782.2 | GISAID |
| 87 | EPI_ISL_6647956 | GISAID |
| 88 | EPI_ISL_6640919 | GISAID |
| 89 | EPI_ISL_7398681 | GISAID |
| 90 | EPI_ISL_7226262 | GISAID |
| 91 | EPI_ISL_7398758 | GISAID |
| 92 | EPI_ISL_6913953.2 | GISAID |
| 93 | EPI_ISL_7406126 | GISAID |
| 94 | EPI_ISL_7381102 | GISAID |
| 95 | EPI_ISL_7137310 | GISAID |
| 96 | EPI_ISL_7015235 | GISAID |
| 97 | EPI_ISL_3160245 | GISAID |
| 98 | EPI_ISL_6436737 | GISAID |
| 99 | EPI_ISL_11903148 | GISAID |
| 100 | EPI_ISL_11714002 | GISAID |

| | | |
|---|---|---|
| 101 | EPI_ISL_11449567 | GISAID |
| 102 | EPI_ISL_11903137 | GISAID |
| 103 | EPI_ISL_11449566 | GISAID |
| 104 | EPI_ISL_10502776 | GISAID |
| 105 | EPI_ISL_11903146 | GISAID |
| 106 | EPI_ISL_11720066 | GISAID |
| 107 | EPI_ISL_11698114 | GISAID |
| 108 | EPI_ISL_11449569 | GISAID |
| 109 | EPI_ISL_12072505 | GISAID |
| 110 | EPI_ISL_11872693 | GISAID |
| 111 | EPI_ISL_11701180 | GISAID |
| 112 | EPI_ISL_11579173 | GISAID |
| 113 | EPI_ISL_11490679 | GISAID |
| 114 | EPI_ISL_11490660 | GISAID |

**Supplementary Table S2.** The comparison of mean ± SEM Ct values of SARS-CoV-2 gene targets and the internal control of the adjusted real-time RT-PCR assay when the RT time was varied from 5 minutes to 4 minutes, 2 minutes, and 30 seconds using one-way ANOVA with Turkey's multiple comparison test.

| **Target** | **RT Time** | **Mean±SEM** | ***p*-value** |
|---|---|---|---|
| ORF1ab | 5 minutes | 23.201±0.112 | 0.013* |
| | 4 minutes | 22.361±0.112 | |
| | 2 minutes | 22.604±0.227 | |
| | 30 seconds | 22.737±0.048 | |
| ORF3a | 5 minutes | 22.741±0.104 | 0.188 |
| | 4 minutes | 22.905±0.072 | |
| | 2 minutes | 22.950±0.204 | |
| | 30 seconds | 23.176±0.076 | |
| N | 5 minutes | 20.719±0.095 | 0.037* |
| | 4 minutes | 21.143±0.075 | |
| | 2 minutes | 21.475±0.349 | |
| | 30 seconds | 21.080±0.069 | |
| ASBVd | 5 minutes | 22.182±0.044 | 0.0001* |
| | 4 minutes | 22.815±0.060 | |
| | 2 minutes | 23.391±0.236 | |
| | 30 seconds | 23.519±0.165 | |

*$p$ value < 0.05

**Supplementary Table S3.** The comparison of mean ± SEM Ct values of SARS-CoV-2 gene targets and the internal control of the adjusted real-time RT-PCR assay when varying initial denaturation time from 1 minute to 30 and 2 seconds using one-way ANOVA with Turkey's multiple comparison test.

| **Target** | **Initial Denature Time** | **Mean±SEM** | ***p*-value** |
|---|---|---|---|
| ORF1ab | 1 minute | 23.729±0.133 | 0.001* |
| | 30 seconds | 22.570±0.114 | |
| | 2 seconds | 22.447±0.157 | |
| ORF3a | 1 minute | 23.269±0.090 | 0.855 |
| | 30 seconds | 22.747±0.172 | |
| | 2 seconds | 22.963±0.130 | |
| N | 1 minute | 20.982±0.149 | 0.986 |
| | 30 seconds | 20.993±0.102 | |
| | 2 seconds | 21.014±0.160 | |
| ASBVd | 1 minute | 22.615±0.246 | 0.007* |
| | 30 seconds | 24.286±0.285 | |
| | 2 seconds | 23.749±0.165 | |

*$p$ value < 0.05

**Supplementary Table S4.** The comparison of mean ± SEM Ct values of SARS-CoV-2 gene targets and the internal control of the adjusted real-time RT-PCR assay when cycling denaturation and annealing/extension time were compared between 2 seconds-4 seconds and 1 second-1 second using unpaired *t*-test.

| Target | Denaturation and Annealing/Extension Time | Mean±SEM | *p*-value |
|---|---|---|---|
| ORF1ab | 2 seconds and 4 seconds | 23.761±0.118 | 0.100 |
| | 1 second and 1 second | 20.684±0.091 | |
| ORF3a | 2 seconds and 4 seconds | 23.127±0.086 | 0.100 |
| | 1 second and 1 second | 22.202±0.130 | |
| N | 2 seconds and 4 seconds | 21.023±0.062 | 0.100 |
| | 1 second and 1 second | 20.346±0.092 | |
| ASBVd | 2 seconds and 4 seconds | 22.469±0.315 | 0.100 |
| | 1 second and 1 second | 23.736±0.075 | |

**Supplementary Table S5.** The comparison of mean ± SEM Ct values of SARS-CoV-2 gene targets and the internal control of the adjusted real-time RT-PCR assay when varying annealing/extension temperatures from 60°C to 65°C using one-way ANOVA with Turkey's multiple comparison test.

| **Target** | **Temperature (°C)** | **Mean±SEM** | ***p*-value** |
|---|---|---|---|
| ORF1ab | 60 | 20.684±0.091 | <0.0001* |
| | 61 | 20.550±0.063 | |
| | 62 | 21.031±0.112 | |
| | 63 | 21.772±0.492 | |
| | 64 | 21.995±0.186 | |
| | 65 | 24.596±0.291 | |
| ORF3a | 60 | 22.202±0.130 | 0.136 |
| | 61 | 22.167±0.073 | |
| | 62 | 22.545±0.045 | |
| | 63 | 22.867±0.390 | |
| | 64 | 22.259±0.066 | |
| | 65 | 22.360±0.149 | |
| N | 60 | 20.346±0.092 | 0.167 |
| | 61 | 20.106±0.071 | |
| | 62 | 20.430±0.100 | |
| | 63 | 20.896±0.491 | |
| | 64 | 20.430±0.140 | |
| | 65 | 20.878±0.150 | |
| ASBVd | 60 | 23.736±0.075 | 0.002* |
| | 61 | 23.597±0.233 | |
| | 62 | 24.515±0.281 | |
| | 63 | 25.996±1.282 | |
| | 64 | 25.318±0.357 | |
| | 65 | 28.409±0.689 | |

*$p$ value < 0.05

**Supplementary Table S6.** The comparison of mean ± SEM Ct values of SARS-CoV-2 gene targets and the internal control of the adjusted real-time RT-PCR assay when varying denaturation temperatures from 82 to 85, 88, 90, and 92°C using one-way ANOVA with Turkey's multiple comparison test.

| **Target** | **Temperature (°C)** | **Mean±SEM** | ***p*-value** |
|---|---|---|---|
| ORF1ab | 82 | 34.422±2.741 | <0.0001* |
| | 85 | 21.128±0.089 | |
| | 88 | 21.007±0.095 | |
| | 90 | 21.274±0.104 | |
| | 92 | 20.928±0.050 | |
| ORF3a | 82 | 30.638±1.977 | 0.0002* |
| | 85 | 22.214±0.091 | |
| | 88 | 22.316±0.144 | |
| | 90 | 22.494±0.090 | |
| | 92 | 22.177±0.023 | |
| N | 82 | 27.982±1.585 | <0.0001* |
| | 85 | 20.364±0.017 | |
| | 88 | 20.444±0.116 | |
| | 90 | 20.709±0.113 | |
| | 92 | 20.521±0.067 | |
| ASBVd | 82 | 27.565±0.309 | 0.0024* |
| | 85 | 25.604±0.018 | |
| | 88 | 25.863±0.345 | |
| | 90 | 26.304±0.318 | |
| | 92 | 25.615±0.169 | |

*$p$ value < 0.05

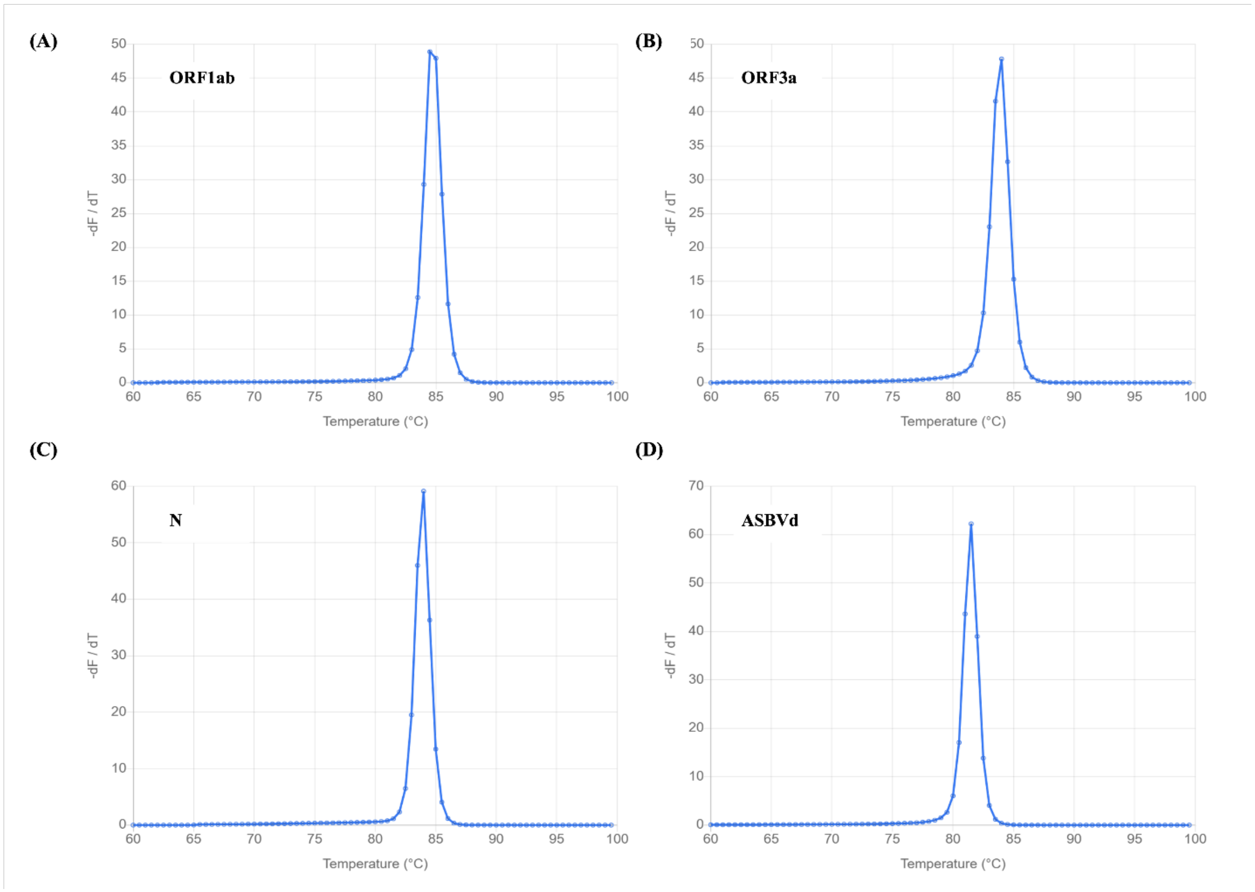


**Supplementary Figure S1.** The predicted melting temperature of each amplicon. The melting temperature (Tm) of each amplicon was predicted using uMelt Quartz. Tm curve of the amplicons **(A)** ORF1ab, **(B)** ORF3a, **(C)** N, and **(D)** the internal control (ASBVd).

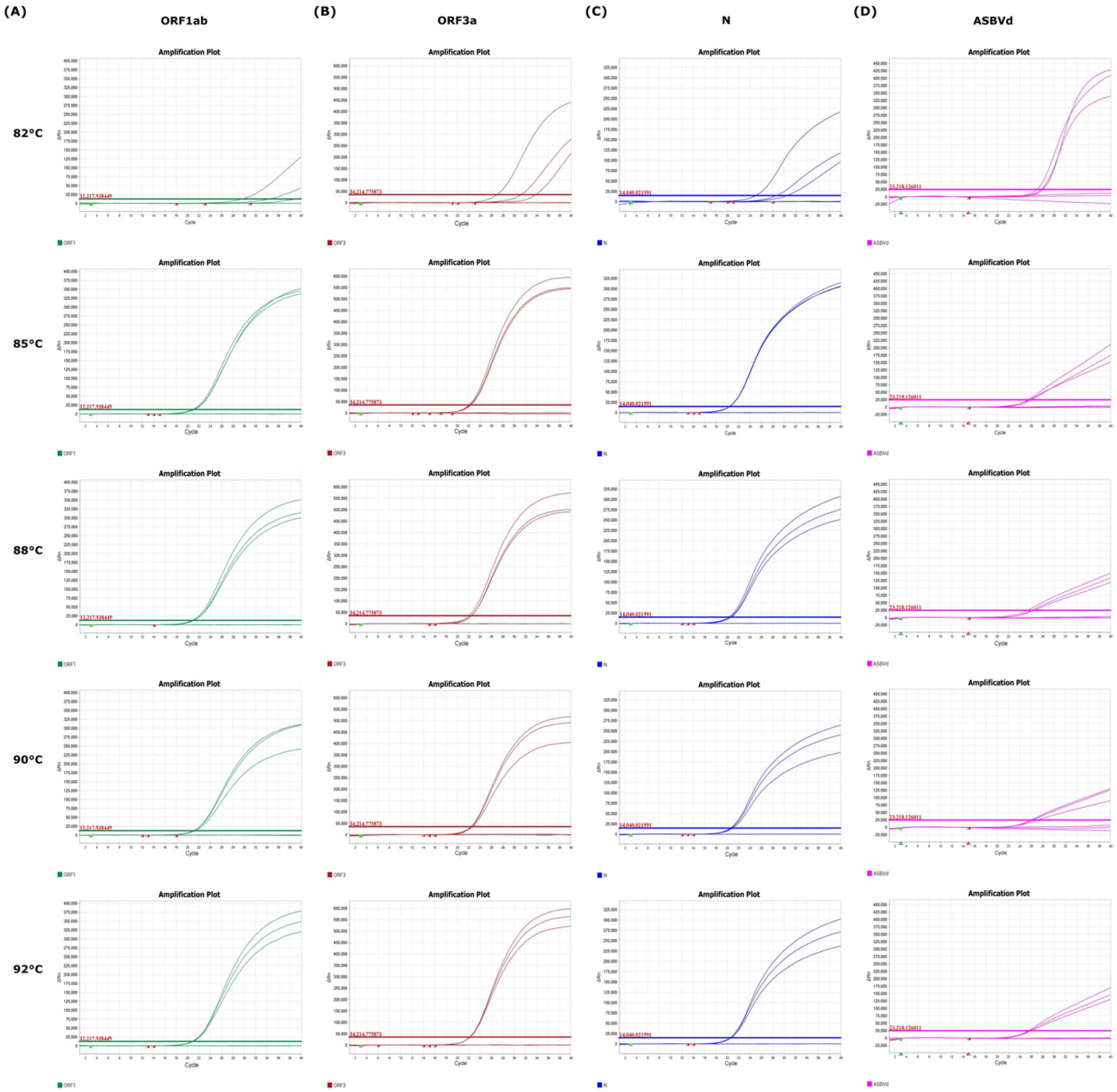


**Supplementary Figure S2.** The amplification plots of SARS-CoV-2 gene targets (ORF1ab, ORF3a, and N genes) and the internal control (ASBVd) among different denaturation temperatures. The denaturation temperature of the real-time RT-PCR assay was varied from 82 to 85, 88, 90, and 92°C. Forty cycles of amplification were done in triplicate for each setting.

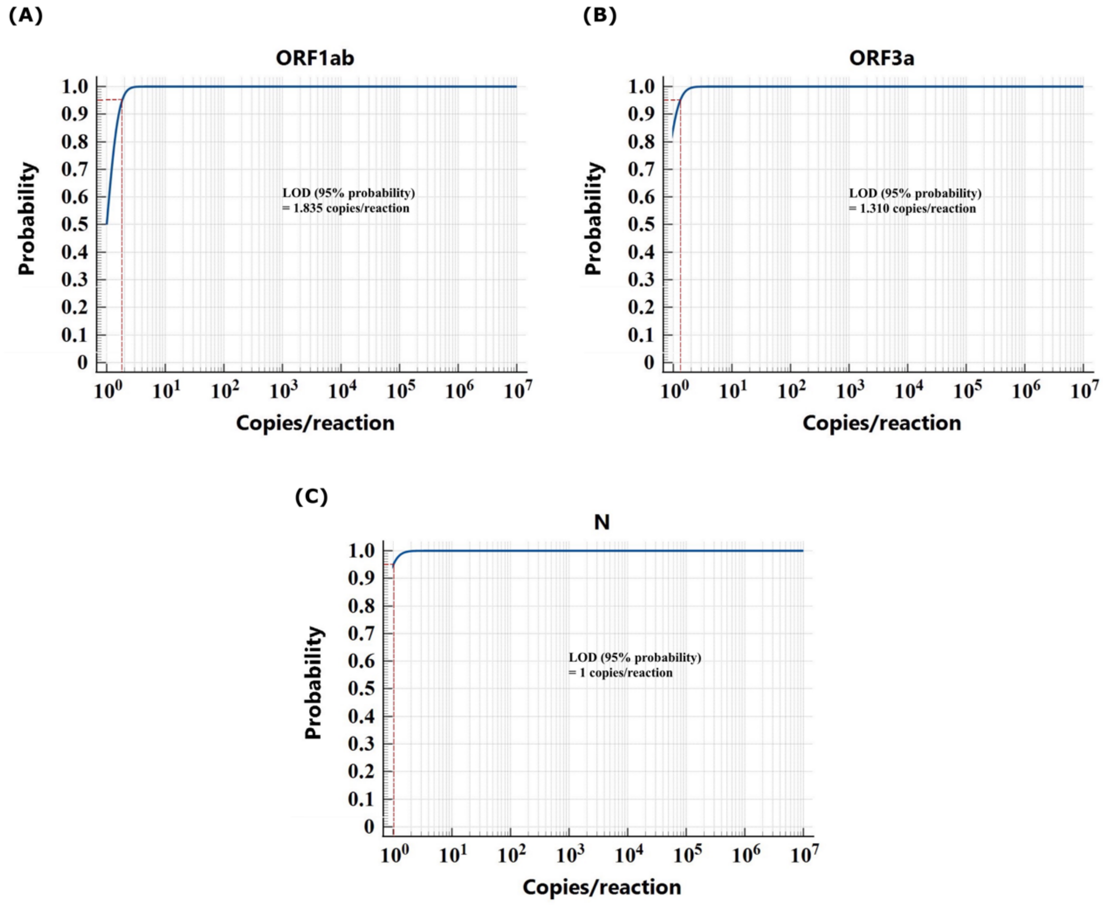


**Supplementary Figure S3.** The limit of detection (LOD) of the rapid real-time RT-PCR assay (ΣS COVID-19). The LOD of each SARS-CoV-2 gene target (ORF1ab, ORF3a, and N genes) was calculated using probit regression analysis at the 95% probability level based on 20 replicates of the reference SARS-CoV-2 RNA ranging from $10^6$ to 1 copies/reaction. The probit analysis of the **(A)** ORF1ab, **(B)** ORF3a, and **(C)** N genes of the SARS-CoV-2.
